# Identifying group metacognition associated with medical students’ teamwork satisfaction in an online small group tutorial context

**DOI:** 10.1186/s12909-024-06116-4

**Published:** 2024-10-09

**Authors:** Chia-Ter Chao, Yen-Lin Chiu, Chiao-Ling Tsai, Mong-Wei Lin, Chih-Wei Yang, Chiao-Chi Ho, Chiun Hsu, Huey-Ling Chen

**Affiliations:** 1 Division of Nephrology, Department of Internal Medicine, National Taiwan University Hospital and National Taiwan University College of Medicine, Taipei, Taiwan; 2 Division of Nephrology, Department of Internal Medicine, Min-Sheng General Hospital, Taoyuan City, Taiwan; 3 Graduate Institute of Toxicology, National Taiwan University College of Medicine, Taipei, Taiwan; 4 Center of Faculty Development, National Taiwan University College of Medicine, Taipei, Taiwan; 5 Graduate Institute of Medical Education and Bioethics, National Taiwan University College of Medicine, No. 1, Sec 1, Ren-Ai Road, Taipei, 100 Taiwan; 6 Division of Radiation Oncology, Department of Oncology, National Taiwan University Hospital, Taipei, Taiwan; 7 Department of Surgery, National Taiwan University Hospital, Taipei, Taiwan; 8 Department of Emergency Medicine, National Taiwan University Hospital, Taipei, Taiwan; 9 Division of Chest Medicine, Department of Internal Medicine, National Taiwan University Hospital, Taipei, Taiwan; 10 Department of Medical Oncology, Department of Medical Education & Research, National Taiwan University Cancer Center, Taipei, Taiwan; 11 Department of Pediatrics, National Taiwan University Hospital, Taipei, Taiwan

**Keywords:** Medical education, Metacognition, Online collaborative learning, Regulation skills, small group tutorial

## Abstract

**Background:**

Collaborative learning is an essential pedagogy in medical education, within which small group learning constitutes an integral component. Online small group teaching has been widely applied and blended with in-person sessions in the aftermath of the Covid-19 pandemic. This study examined whether group metacognition was associated with teamwork satisfaction in an online small group teaching curriculum for medical students.

**Methods:**

We enrolled medical students of the 2nd and 4th years during the 2021 fall semester after they participated in 3 consecutive sessions of online small group tutorials (SGTs), which have been implemented in our medical school for more than 20 years. The students completed a group metacognitive scale (GMS) and a teamwork satisfaction scale (TSS) after the sessions. We analyzed whether group metacognition in 4 dimensions (knowledge of cognition, planning, evaluating, and monitoring) could be connected with medical students’ teamwork satisfaction using partial least squares-structural equation modeling (PLS-SEM).

**Results:**

A total of 263 medical students participated in this study. Both GMS and TSS exhibited good reliability and validity. Three of the 4 dimensions of group metacognition (cognition, planning, and evaluating) positively correlated with teamwork satisfaction (path coefficients 0.311, 0.279, and 0.21; *p* = 0.002, 0.002, and 0.043, respectively) following the online SGT curriculum, whereas the monitoring dimension did not (path coefficient 0.087; *p* = 0.357). The model achieved an adjusted R square of 0.683.

**Conclusion:**

We discovered that group metacognition correlated positively with better teamwork satisfaction, supporting the importance of group metacognitive competency for online collaborative learning.

**Supplementary Information:**

The online version contains supplementary material available at 10.1186/s12909-024-06116-4.

## Background

### Metacognition: importance in higher education

Metacognition refers to the awareness of one’s learning status, including phases of planning, monitoring, and evaluation, and the ability to monitor and regulate one’s cognitive process, a core motivating force for learners to understand their own abilities [1, 2]. Learners with better metacognitive competence or greater familiarity with metacognitive strategies may have better learning motivation and outperform those without such ability, especially in higher education [3, 4]. Theoretically, metacognitive learning allows learners to manage their own growth and foster autonomous learning, a key ability in the contemporary medical education which requires continuous uptake of medical advancement and new technologies [1]. Targeting metacognition for cultivation or improvement can enhance students’ learning motivation and academic performance [5].

### Metacognition in medical education

Metacognitive strategies are highly useful within learning contexts in medical education, and may supplement other medical education pedagogies such as competency-based medical education to enhance self-learning [6]. Hong et al. revealed that better metacognitive competence correlated moderately with academic performance among medical students [7]. High academic performers have better metacognitive awareness compared to low and middle performers in undergraduate medical education [8]. Recent studies further evaluate whether metacognition may enhance students’ learning performance and outcomes in online learning, particularly for undergraduate students during the pandemic period. A prior study showed that metacognitive skills significantly assisted college students in online lectures, whereas the lack of such skills placed students at a disadvantage [9]. We were interested in looking into whether metacognitive skills correlate with student performance in other online curricula, especially those involving teamwork.

### Online small group tutorials: Interaction counts

Collaborative learning, especially small group teaching, can enhance cognitive skills, communication, and self-directed learning, leading to its rising popularity over time. Small group teaching further empowers learners to formulate their coping strategies with perceived barriers [10, 11]. Our school has developed a dedicated curriculum, Small Group Tutorials (SGT), to facilitate the integrated learning of basic medical science, clinical medicine, and medical humanities in undergraduate medical education [12–14].

Online small group teaching has received enormous attention since the coronavirus pandemic. The employment of an online setting not only circumvents the risk of person-to-person pathogen transmission, but also renders the curricular schedule more flexible. However, bridging the gap between in-person and online curricula can be troublesome and requires more preparation beforehand [15]. More importantly, the online interactions between participants, or the team dynamics, differ from those in face-to-face settings. Students in online small-group teaching rate “communication and interaction” as the most pivotal theme influencing their collaborative learning efforts [16]. The initiation of interaction during online collaborative learning and socialized connections correlates significantly with learning outcomes [17]. Furthermore, interactions between participants influence learners’ perceptions of the curriculum, suggesting the importance of teamwork experiences and how well participants are satisfied with team processes. How to improve team processes and increase learners’ enjoyment in team processes has subsequently emerged as a vital issue of small group teaching.

### Satisfaction with online learning

Student satisfaction, a fundamental component for evaluating the quality of online learning, has been considered an essential predictor to assess the effects and sustainability of online learning programs [18]. It has been revealed that students’ experiences with online learning can be hindered by factors such as information overload and perceived skill requirements [19]. In addition, technical skills and tutor support are important factors correlated to learners’ satisfaction in online modules [20]. A scoping review of 53 studies showed that communication dynamics (e.g., information quality and interactions), environmental factors (e.g., course structure, ease of use, and usefulness), organizational factors (e.g., service quality, organizational support), and personal factors (e.g., age, gender, skills, and prior experience) were critical predictive factors of satisfaction and perceived learning outcomes in online environments. Another review revealed that medical students’ satisfaction with online learning was correlated with demographic factors, prior experiences with online learning, and course arrangement details [21]. In spite of the fact that existing studies have investigated students’ satisfaction with online courses, insufficient importance is given to students’ prerequisites such as readiness, self-regulated skills and cognitive requirements for online learning contexts [22]. Teamwork satisfaction during online small-group teaching may therefore serve as an independent endpoint worthy of further investigation.

### Metacognition in online learning environments

Online self-regulation involves the acquisition and utilization of cognitive processes that enable learners to effectively plan and organize their learning tasks within online environments [23]. While concerning successful online education, students’ regulation skills are regarded as contributing factors which improve their online learning satisfaction and lead to greater effects on online education [22, 24]. Metacognitive knowledge (i.e., understanding about cognition) and metacognitive regulation processes (i.e., utilization of that knowledge to regulate cognition) are defined as two major components of metacognition [25]. In online group-based learning contexts, metacognition has been conceptualized as knowledge of cognition as well as regulatory skills such as planning, monitoring and evaluating in online collaborative learning processes [26]. Metacognition has been considered an essential competency and has been extensively explored in online learning environments to enhance learner engagement [27], and to facilitate problem-based learning with online technology and tools [28, 29], particularly within online small group discussion contexts [30].

### Group metacognition in collaboration

Group metacognition, underpinned by social constructivism, is conceptualized as the ability to reflect on the group’s cognitive skills during collaboration. This includes awareness of members’ decision-making, information organization, planning, revising, improving, and assessing group work processes [26, 31]. Analysis of group discourse suggests that meta-level discourse about the group’s process, rather than individuals’ actions regarding metacognitive skills, is associated with superior group outcomes [32]. It has been demonstrated that higher levels of group metacognition lead to better collaboration during the problem-solving process [31]. Since traditional teamwork studies have focused on individual metacognitive skills, this study builds on earlier research by emphasizing group metacognition and the importance of regulated social behavior in online collaborative settings.

Stronger metacognition is linked to higher academic achievement and clinical competency, suggesting the promotion of metacognitive skills in individual learning processes [8, 33]. While most research has focused on individual learners, some has suggested that students in small groups can enhance their mutual metacognition, leading to better learning outcomes [34]. Metacognition has primarily been studied individually, whilst neglecting the importance of group-regulated behavior during cooperative activities and how group members perceive and reflect on their collective skills [26]. However, group metacognition is a crucial component for successful collaborative learning [31, 35]. Metacognition used in collaborative learning focuses on reflecting on the group’s cognitive status, known as group metacognition, which benefits individuals while providing additional advantages for teamwork [26].

Effective team performance relies on sufficient situation awareness for positive teamwork, emphasizing the importance of mutual performance monitoring and metacognition in cooperation [36]. Research has highlighted the importance of group metacognition and regulated social behavior in collaboration, showing that individual regulation alone is insufficient for social learning and that collaborative activities positively impact students’ group metacognition [31]. Measuring unobservable aspects in collaborative learning contexts, such as mutual performance monitoring, has been emphasized. Existing studies stress that enhancing metacognitive awareness in cooperation can significantly improve team performance [26, 36].

### Study hypotheses

**Fig. 1:**
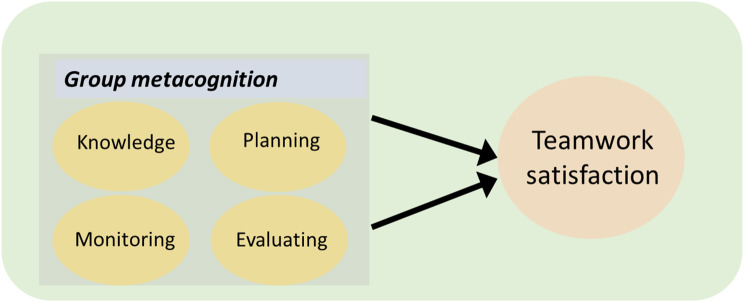
Our research framework

The expanded application of group metacognition to teamwork has educational merits. Harnessing metacognitive strategies during teamwork settings such as multidisciplinary care and in undergraduate online learning may enhance professional competence, reduce misunderstandings, and increase learning efficacy [9, 37]. However, it remains unclear how group metacognition can be connected to students’ teamwork experiences, which is an independent yet under-appreciated endpoint, as explained above, during online small-group learning. We hypothesized that better group metacognition might enhance teamwork satisfaction among medical students in an online SGT curriculum (Fig. 1). This study therefore aimed at identifying key dimensions of group metacognition associated with teamwork satisfaction to guide further curricular improvement.

## Materials and methods

### Ethical statement

This study was approved by the National Taiwan University Hospital ethical review board (NO. 202108011RIND). The ethical board waived the need for written informed content from participants due to the anonymized process of data collection used in this study and the perceived minimal risk of participant harm. The performance of this study adhered to the Declaration of Helsinki.

### Study procedure and participant enrollment

In this study, the curricular setting was SGT for 2nd and 4th year medical students. National Taiwan University College of Medicine (NTU-CM) has adopted SGT curriculum for more than two decades, with the core concept of teachers/tutors as learners. Face-to-face SGT in NTUCM is a collaborative learning style curriculum, with the following features. Participants included six to ten 2nd to 4th grade medical students per group, to which one tutor was assigned. During the once weekly course, clinical cases and pre-designed questions are provided to each group, with tutor- or student-generated new questions spurring discussions. Students need to undertake pre-session task assignment, participate in discussion during each session, and tutors can provide post-session feedback and wrap-up. In response to the coronavirus disease 2019 (COVID-19) pandemic, the NTU-CM started its online SGT curriculum in 2020 and has continuously improved the platform, the infrastructure, and the content of online SGT based on feedback from the administrative office, tutors, coordinators, and participants. During the online SGT curriculum, students are divided into small groups with 6–10 members facilitated by one physician of diverse disciplines. Each group takes part in a weekly discussion of 2 h length, focusing on one clinical case and scenarios (medical humanity and patient-physician communication, with focuses on pharmacology and pathology) drafted by volunteer physicians. Tutors aim to inspire students to integrate basic science, clinical medicine, and medical humanity issues. Students are given opportunities to prepare for discussion beforehand, whereas tutors can choose to provide feedback on unresolved issues or group performance after each session.

Medical students of the 2nd and 4th year from NTU-CM, aged between 20 and 23 years, were prospectively enrolled during their fall semester in 2021. Prior to course initiation, we explained about this study, which aimed to continuously improve our teaching quality and jointly shape the next generation of medical education through reciprocal feedback from medical students after online SGT. Joining this study and providing responses did not influence participants’ grade, which was rated by tutors of each group independent of study investigators. After participating in three consecutive online SGT sessions, medical students were instructed to complete the study instruments at the end of the last session. Each student completed his/her own surveys without interferences from peers, using the REDCAP software, an online anonymized computer-generated feedback system created by our institute. Students of each group contributed to the results from the group perspective.

### Study instruments

We employed two instruments in this study, a group metacognition scale (GMS) and a teamwork satisfaction scale (TSS) (Supplementary Table S1). The former was adapted from an existing instrument [26], which was created to measure group metacognition during online collaborative learning activities. GMS consisted of 20 items allocated to four dimensions (knowledge of cognition, planning, monitoring, and evaluating; 5 items each), rated on a Likert-type scale (1 to 5, with higher numbers indicating greater concurrence). Knowledge of cognition denotes the level to which an individual is aware of their cognition during the group process, whereas planning dimension assesses how team members allocate their resources and select proper approaches to optimize group performance. Samples of knowledge of cognition and planning dimension are “We know our strengths as learners…” and “We determine what the task requires…,” respectively. The monitoring dimension measures team members’ awareness of group task comprehension and the self-instruction process. The evaluating dimension involves the critical appraisal of the group process, aiming to reflect on collaborative learning. Samples of monitoring and evaluating dimensions were “We check our approach to improve our outcomes…” and “We make judgments on the workload…,” respectively. GMS was validated based on a group of university students [26]. The wording of each item was examined to reassure context appropriateness with our SGT setting, followed by translation into traditional Chinese. The translated version was further checked by faculty of the Institute of Medical Education and Bioethics in NTU-CM and senior SGT tutors to ensure wording accuracy.

TSS was originally developed by Tseng et al. [38], and consists of 10 items rated on a 5-point Likert-type scale. TSS is a self-administered, uni-dimensional instrument focusing on team members’ satisfaction with the teamwork learning environment and interactions. TSS encompasses three perspectives; satisfaction with the environment of online collaborative learning, the process of teamwork learning, and the merits originating from group interactions. A sample TSS item is, “I like to work in a collaborative group with my teammates.” TSS was designed and then validated with graduate students participating in online collaborative learning activities [38, 39].

### Statistical analysis

We analyzed whether group metacognition in different dimensions influenced medical students’ teamwork satisfaction using partial least squares-structural equation modeling (PLS-SEM) as suggested previously by the analytic guidance of SEM [40]. For the PLS-SEM approach, we first examined the factor loadings, average variance explained, composite reliability, and the Fornell-Lacker test to ensure the reliability and validity of each instrument. Items of the GMS and TSS exhibiting inadequate estimates were removed to improve the model fitness [41]. We next verified SEM with path analyses to investigate the relationship between the four dimensions of GMS and teamwork satisfaction, with path coefficients provided. In all analyses, a *p* value < 0.05 was deemed statistically significant during interpretation. We used the SmartPLS3 software to carry out the PLS-SEM analyses.

## Results

A total of 263 medical students of 2nd and 4th grade participated in this study, after completing three consecutive sessions of online SGT. They completed the GMS and TSS surveys, and the results were directly exported from the REDCAP system for subsequent analysis.

### PLS-SEM analysis for the measurement model

Following the PLS analysis, we found that two items of the GMS scale, one from the evaluating dimension and the other from the monitoring dimension, had low loadings (< 0.5) and were therefore removed. Other item loadings are provided in Table 1. The remaining four dimensions with 18 items constituted a reasonable measurement model, with a composite reliability ranging between 0.91 and 0.94, indicating good reliability (Table 1). The average variance extracted lay between 0.67 and 0.79, suggesting that our model had acceptable convergent validity. The internal consistency, assessed by Cronbach’s alpha, was excellent (from 0.87 to 0.92). Using the Fornell-Larcker test, we showed that the square roots of average variance for each dimension were higher than the inter-factor correlation coefficients (Table 2), supporting the fair discriminant validity of our measurement model.

For TSS, one item was removed for low factor loading, leaving nine items, with loadings provided in Table 1. The composite reliability was 0.95, whereas the average variance extracted was 0.69. The discriminant validity of the TSS was also adequate (Table 2).

**Table 1:** Validity and reliability analyses of the group metacognition scale and teamwork satisfaction scale

Dimensions/items	Loading	Composite reliability	Average variance extracted	Rho value	α value
**Group metacognition scale**					
*Knowledge of cognition*		0.937	0.748	0.917	0.916
K1	0.843				
K2	0.892				
K3	0.862				
K4	0.894				
K5	0.832				
*Planning*		0.909	0.666	0.876	0.874
P1	0.771				
P2	0.852				
P3	0.829				
P4	0.845				
P5	0.779				
*Evaluating*		0.939	0.794	0.916	0.914
E1	0.855				
E2	0.899				
E3	0.893				
E4	0.918				
*Monitoring*		0.936	0.786	0.92	0.909
M1	0.832				
M2	0.888				
M3	0.908				
M4	0.916				
**Teamwork satisfaction**					
*Satisfaction*		0.952	0.686	0.943	0.942
S1	0.879				
S2	0.818				
S3	0.876				
S4	0.849				
S5	0.825				
S6	0.823				
S7	0.843				
S8	0.73				
S9	0.802				

GMS, group metacognition scale; TWS, teamwork satisfaction scale

**Table 2:** Discriminant validity analysis results

Dimensions	Planning	Evaluating	Knowledge of cognition	Monitoring	Teamwork Satisfaction
Planning	***0.816***				
Evaluating	0.811	***0.891***			
Knowledge of cognition	0.829	0.824	***0.865***		
Monitoring	0.829	0.875	0.817	***0.886***	
Teamwork Satisfaction	0.778	0.768	0.786	0.755	***0.828***

### Path coefficients of structural equation modeling

We then used the PLS-SEM model to analyze the path coefficients between dimensions of group metacognitive competence and teamwork satisfaction among participating medical students. In our model, the variance inflation factors for independent variables ranged between 1.79 and 4.54, suggesting the absence of collinearity. The standard root mean square residual (SRMR) and normed-fit index (NFI) of the PLS-SEM results were 0.05 (< 0.08) [42] and 0.83 (> 0.8) [43], respectively, indicating acceptable model fitness.

**Fig. 2:**
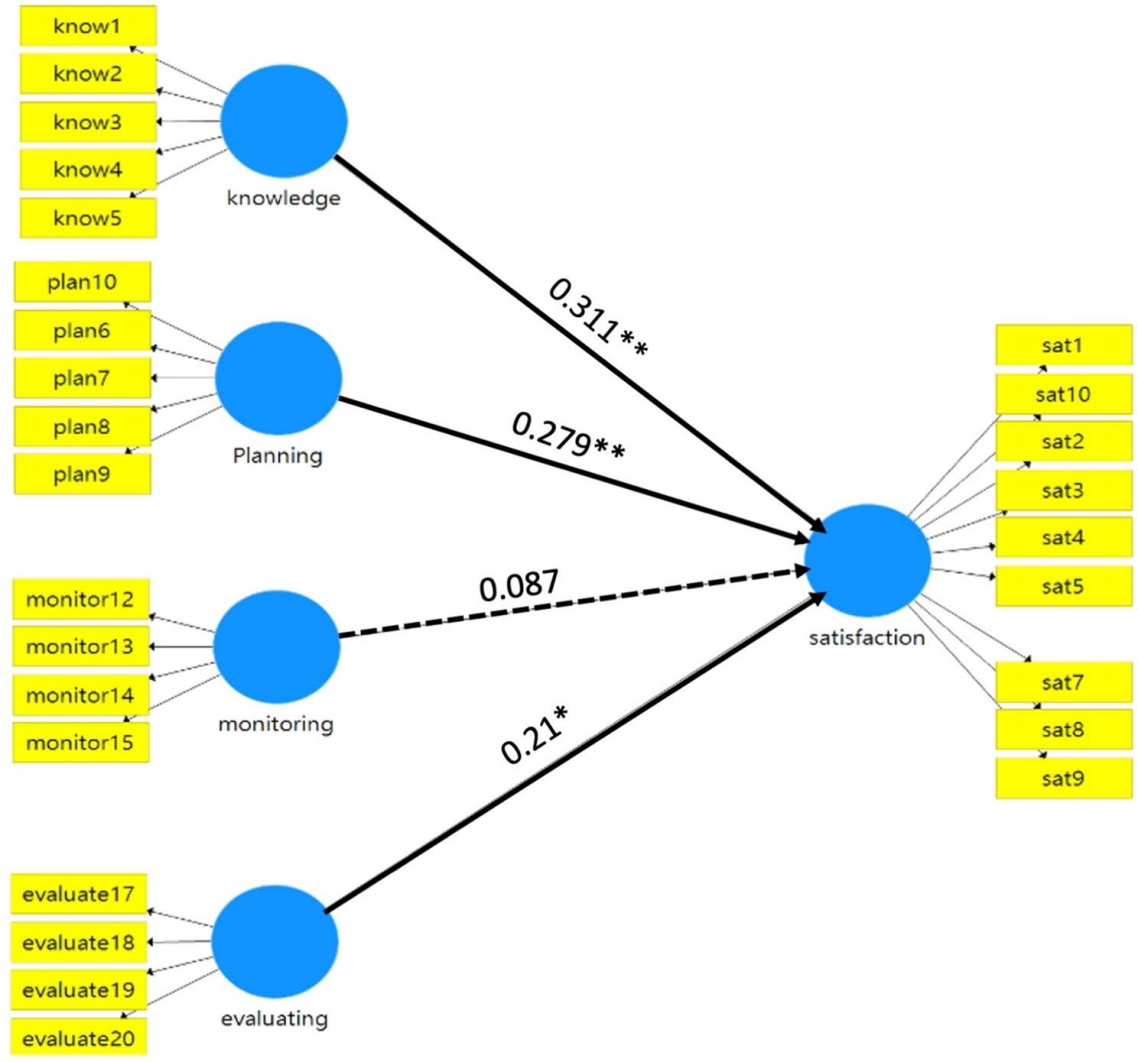
The partial least squares-structural equation modeling results for the measurement and the structural model. Numbers above the arrow indicate path coefficients. *p < 0.05; **p < 0.01. Solid lines represent a significant path coefficient, while the dashed line represents a non-significant path coefficient. *know: knowledge; sat: satisfaction*

The path coefficients and relationships are shown in Fig. 2; Table 3. Among the four dimensions of metacognition competence, knowledge of cognition, planning, and evaluating had a positive correlation (path coefficient 0.31, 0.28, and 0.21, *p* = 0.002, 0.002, and 0.043, respectively) with teamwork satisfaction following the online SGT courses. However, the monitoring dimension did not correlate with teamwork satisfaction (*p* = 0.357) (Table 3). In our analyses, the R square value was 0.688, and the adjusted R square value was 0.683.

**Table 3:** Path analysis results

Outcome: teamwork satisfaction			
	Path coefficients	*t*	*p* value
Knowledge of cognition	0.311	3.056	*0.002*
Planning	0.279	3.186	*0.002*
Monitoring	0.087	0.921	*0.357*
Evaluating	0.21	2.033	*0.043*

R square = 0.688; Adjusted R square = 0.683

## Discussion

In this study, we found that knowledge of cognition, planning, and evaluating were independently associated with better teamwork satisfaction among medical students in the online SGT curriculum, whereas the monitoring dimension was not. The findings support our hypothesis that improving group metacognition may enhance teamwork satisfaction in online SGT courses.

Prior literature concerning the educational importance of metacognition mostly centered on individual learning [3, 5, 44], while relatively few studies addressed the role of metacognition on teamwork-based learning scenarios. Clinical teaching frequently occurs as a collective learning process, in which trainees learn how to gather information, interact with peers, members of different professions and their supervisors, and reach conclusions with the entire medical team. In undergraduate education, team learning also plays an important role, as curricular requirements are often only attainable through teamwork, and collaborative learning is a prevalent education platform. Learning and practice as a team should also be a priority for lifelong learners, especially medical students. It is therefore vital to uncover instrumental factors for optimizing teamwork experiences, so as to maintain or even stimulate students’ interest in learning with or as a team. Upholding the metacognitive competency of collaborative teams appears to be one of the candidates, based on our findings.

Multiple dimensions of group metacognition appear to positively contribute to teamwork satisfaction, including knowledge of cognition, planning, and evaluating (Table 3). According to the situated learning model [45], learning is a social process incorporating the environment and sociocultural context in which it occurs. The understanding of this process and the subsequent prompting of communication solidifies the knowledge the learners are capturing, especially during group learning. Consequently, the awareness of such requirements and maintaining the interactive process can potentially assist in knowledge retention, teamwork effectiveness, and greater enjoyment of the process. Knowledge of cognition thus exhibits a tight correlation with teamwork satisfaction. Furthermore, in our SGT curriculum, the materials were clinical cases with descriptions and quizzes, requiring the integration of prior knowledge, clinical clues, and the local healthcare infrastructure for problem-solving [12, 14]. Content was frequently domain-specific, necessitating pre-curricular preparation for curating details and extracting case themes, particularly during the online small group teaching course. Students who were trained to use metacognitive strategies, especially planning and evaluating, had prominent improvements in their reading and comprehension ability [46]. The internalization of curricular materials before small group teaching can strengthen team processes and possibly enhance satisfaction. In addition, during our online SGT, group participants may encounter conflict between members and divergence of learning goals during discussion, partially due to the absence of cues available during in-person discussion [47]. The awareness of such probability, constantly evaluating the team process, and managing difficult conditions during discussion likely improve course smoothness and increase satisfaction. Critical thinking can originate from these metacognitive strategies and help learners to upgrade their subsequent learning experiences [48]. It is thus reasonable that the metacognitive dimensions of knowledge of cognition, planning, and evaluating are correlated with better teamwork satisfaction.

The lack of association between the metacognitive dimension monitoring and teamwork satisfaction is intriguing. Monitoring within metacognitive competence refers to the ability to accurately monitor one’s learning process based on attention focusing, self-instruction, and self-coaching [49]. Information harvested during the self-monitoring process is then compared with the pre-set learning goal, followed by self-evaluation and reactions. Several reasons may be responsible for this phenomenon. In our SGT sessions, medical students are expected to focus their attention on extracting useful information from participants’ reports, which may assist them in providing appropriate responses and invigorate the discussion. However, these monitoring actions take time, but may be compressed in the online setting due to technical issues and speech jamming resulting from insufficient stewardship [14]. It is plausible that the involuntary shortening of the monitoring period partially accounts for this lack of relationship with teamwork satisfaction. In addition, a prior study showed that participants in online discussions tend to contribute shorter responses and uniform answers to queries compared to face-to-face discussions, in which there is heterogeneity in responses [50]. The truncated and monotonous messages during online SGT sessions may also contribute to poor monitoring among group members, leading to perceived poor teamwork processes. In addition, metacognitive monitoring can be task-dependent, which may weaken its association with task satisfaction, although the GMS scale we adapted aligned well with the current educational context. Finally, there is a possibility that tutors insufficiently encourage monitoring and communications during online small group teaching, causing participants to rate their interaction process poorly. The online discussions of medical humanity topics may further compromise monitoring. Creative arts and narrative understanding, two crucial tools for medical humanity education [51], might not thrive in online settings as they can in face-to-face settings [52]. Therefore, allocating sufficient time for introspection and attention focusing, aided by appropriate tutor guidance and polishing digital literacy, may contribute to better metacognitive competence within our online SGT curriculum. This directs to finding new approaches to enhance participants’ self-monitoring ability.

Based on our findings, sharpening metacognitive competence for promoting learning efficacy among medical students becomes instrumental, since improving metacognition correlates with better academic performance [7]. The timing of metacognitive ability training is important; metacognitive skill learning should start early during the curriculum to obtain the greatest effect [8]. Explicit teaching of metacognition through reflection and intentional questioning, integrating metacognitive training into routine curricula, and creating a suitable environment, regardless of whether it is in-person or virtual, can be harnessed for this purpose [53, 54]. However, in the collaborative learning contexts such as online SGTs, an individual’s metacognition is insufficient to explain learning in social settings; the positive effects of collaborative activities on students’ group metacognition are also important considerations [26, 34]. Socially shared regulation during collaborative learning may result in social group regulation and gain metacognition [55], suggesting that through instruction which helps team members be aware of how they cooperate with each other to complete team tasks can significantly improve team performance [36]. For example, online scaffolding peer-questioning is helpful for increasing the frequency of social interactions and discussions, and may improve the monitoring and comprehension of the group’s learning task [30].

Our study has its strengths and limitations. It is one of the first studies to focus on group metacognition and its correlation with teamwork satisfaction in an online SGT curriculum. Findings from this study can assist upcoming researchers in devising effective strategies to make online collaborative learning more enjoyable. Furthermore, we identified certain metacognitive dimensions that minimally affected teamwork satisfaction, which warrants further attention. However, several other issues in this study need pondering as well, constituting its limitations. First, our results might not be fully applicable to in-person SGTs or other education platforms. Second, there might be other unmeasured factors influencing teamwork satisfaction in our curriculum (e.g. technical problems). Thirdly, we did not document participants’ digital literacy and information related to their social interactions. Fourthly, we did not measure participants’ individual metacognitive competence, whose correlation with the group metacognition level remained undetermined. Finally, the concept of group metacognition may not be easily distinguished from social metacognition [56], which is more widely recognized and has its own measurement instruments. Nonetheless, our findings may still inspire future work in elucidating the role of group metacognition in SGTs.

## Conclusion

In this study, we surveyed medical students participating in an online SGT, regarding their group metacognition during teamwork and their teamwork satisfaction levels. We employed PLS-SEM for assessing the association between different group metacognitive dimensions and teamwork satisfaction, and discovered that group metacognition in knowledge of cognition, planning, and evaluating were intimately associated with greater teamwork satisfaction, whereas dimension monitoring was not. Findings from this study support the notion that group metacognition contributes to teamwork satisfaction in online collaborative learning, which is an important issue but is rarely discussed. Future efforts to enhance group metacognition may be worth investigation to improve medical education in online learning contexts.

## Electronic supplementary material

Below is the link to the electronic supplementary material.

Supplementary Material 1

## Data Availability

The data that support the findings of this study are not openly available due to reasons of sensitivity and are available from the corresponding author upon reasonable request.

## Abbreviations

GMS: Group metacognition scale
NFI: Normed-fit index
NTU-CM: National Taiwan University College of Medicine
PLS-SEM: Partial least squares-structural equation modeling
SGT: Small group tutorial
SRMR: Standard root mean square residual
TSS: Teamwork satisfaction scale
