# Supplementary material for: Identifying group metacognition associated with medical students’ teamwork satisfaction in an online small group tutorial context
Source: BMC Med Educ. 2024 Oct 9;24:1114. doi: 10.1186/s12909-024-06116-4 (PMC11465512; doi:10.1186/s12909-024-06116-4)
Supplement: Supplementary file 1 — Supplementary Material 1 [file 12909_2024_6116_MOESM1_ESM.docx]

**Supplementary Table S1**. The group metacognition scale and teamwork satisfaction scale used in this study

| *Dimensions/items* |
| --- |
| **Group metacognition scale** |
| *Knowledge of cognition* |
| K1. We know our strengths as learners. |
| K2. We know how to select relevant information. |
| K3. We know how to use the material. |
| K4. We know how to organize the new information. |
| K5. We know how to connect new information with prior knowledge. |
| *Planning* |
| P1. We plan the learning activities. |
| P2. We determine what the task requires. |
| P3. We select the appropriate discussion tools. |
| P4. We identify the discussion strategies depending on the task. |
| P5. We organize our time depending on the task. |
| *Evaluating* |
| E1. We make judgments on the workload. |
| E2. We make judgments on the instruments. |
| E3. We make judgments on our learning outcomes. |
| E4. We make judgments on the teamwork process. |
| *Monitoring* |
| M1. We ask questions to check our understanding. |
| M2. We check our approach to improve our outcomes. |
| M3. We improve our work with group processes. |
| M4. We detect and correct errors during group processes. |
| **Teamwork satisfaction** |
| *Satisfaction* |
| S1. I like to work in a collaborative group with my teammates. |
| S2. I like to solve problems with my teammates in group discussions. |
| S3. Interacting with the other team members can increase my motivation to learn. |
| S4. I have benefited from interacting with my teammates. |
| S5. I have benefited from my teammates’ feedback. |
| S6. Online teamwork promotes creativity. |
| S7. Working with my team helps me produce better discussion quality than working individually. |
| S8. My team members are sharing knowledge during the teamwork processes. |
| S9. I gain online collaboration skills from the teamwork processes. |

*GMS, group metacognition scale*
